# Assisted Infant Toilet Training and Bladder and Bowel Health: A Global Integrative Review

**DOI:** 10.1007/s10995-025-04160-0

**Published:** 2025-09-05

**Authors:** Celia Hindmarsh, Deborah Davis, Marjorie Atchan

**Affiliations:** Faculty of Health, University of Canberra, Building 10 B Office 35, University Dr, Bruce, ACT 2617 Australia

**Keywords:** Assisted infant toilet training, Elimination communication, Early toilet training, Bladder and bowel dysfunction, Infant development

## Abstract

**Background:**

Toilet training practices vary across cultures and time. Assisted Infant Toilet Training (AITT) is commonly used in low- and middle-income countries.

**Objectives:**

To synthesise the literature on AITT, including timing of initiation and completion, infant elimination signalling, and associations with bladder and bowel dysfunction.

**Methods:**

An integrative review methodology was employed. Comprehensive searches of Scopus, Medline, CINAHL, Web of Science, PsycINFO, and Google Scholar identified relevant studies. Two reviewers independently screened and appraised studies using GRADE and JBI tools.

**Results:**

Of 2,069 studies identified, 21 met inclusion criteria. Six observational studies reported reduced rates of bladder and bowel dysfunction when AITT was practised.

**Discussion:**

AITT is widely practised in low-income, non-English speaking countries. While observational studies suggest a potential protective effect on bladder and bowel health, the evidence is at serious risk of bias. Further prospective research in high-income contexts is warranted.

## Significance

What is already known about this topic

What this study adds

Toilet training is culturally variable and typically delayed in high-income settings. AITT is widely used in low- and middle-income countries.

This review suggests AITT may protect bladder and bowel health, though stronger evidence is needed; importantly, increased dysfunction was not observed compared to toilet training after 18 months. AITT may reduce an infant’s ecological footprint. The absence of AITT from national toilet training guidelines in many high-income countries appears unjustified.

## Introduction

Toilet training is the process of achieving urinary and faecal continence (Wu, 2010). In high-income countries, toilet training typically begins when an infant is deemed developmentally ready to train independently, usually after eighteen months of age (Horn et al., 2006). Assisted Infant Toilet Training (AITT), also known as Elimination Communication (EC), is an alternative method that shares the same goal as modern toilet training but follows a different timeline and approach (Wu, 2010). AITT is an infant-led, caregiver-assisted practice in which caregivers respond to an infant’s cues and natural timing to recognise elimination needs (Xing et al., 2020). Initiated between birth and 18 months, AITT involves the caregiver teaching the infant to eliminate in a designated receptacle while maintaining a physiologically beneficial squatting position (Jordan et al., 2020). Additionally, caregivers often use cue sounds to help infants associate the elimination process with the relaxation of the pelvic floor and sphincters (Xing et al., 2020). This caregiver-infant partnership has been practised throughout history as a method of maintaining infant hygiene and continues to be widely used in many cultures worldwide.

Cultural shifts and the widespread adoption of disposable diapers have contributed to a significant delay in the toilet training process (Bakker & Wyndaele, 2000; Horn et al., 2006). Toilet training today in high-income countries commonly commences between 21 and 36 months (Horn et al., 2006). The child-oriented toilet training method gained widespread popularity following the work of American pediatrician Terry Brazelton, who published his influential paper on toilet training in 1962 (Brazelton, 1962). Brazelton’s approach encouraged waiting for the child’s signs and signals of readiness, in contrast to the coercive and punitive methods of early toilet training in 1930 s America, which had been criticised for their psychological harm (Accardo, 2006).

The American Academy of Paediatrics (Pediatrics, 2022), the Canadian Paediatric Society and prominent Australian guidelines (Network, 2023) all recommend commencing toilet training after eighteen months based on expert opinion and cultural precedent (Kiddoo, 2012). A systematic review of toilet training methods for infants with normal development from de Carvalho Mrad and colleagues (de Carvalho Mrad et al., 2021) concludes that “*it is not possible to make definitive claims about one method’s superiority over the other*” (de Carvalho Mrad et al., 2021). The omittance of reference to AITT in national toilet training guidelines in many high-income countries appears unjustified.

An integrative review was selected to synthesise knowledge about AITT from experimental and non-experimental sources, present an up-to-date review of this understudied topic and identify research gaps (Whittemore & Knafl, 2005). An overview of the AITT method, including when AITT is initiated and completed, how the infant signals their elimination needs, and if bladder and bowel dysfunctions were encountered, are key thematic outcomes of this review. These outcomes aim to provide an overview of how the method is practised and address concerns about the safety of AITT. Parents, policy writers, and primary health care workers in English-speaking, high-income countries may benefit from understanding the cultural underpinning of toilet training methods worldwide, as the widespread practice of AITT with infants across cultures demonstrates. For those who have never encountered AITT, awareness of AITT can expand their understanding of what babies are capable of, even from birth. Finally, AITT has the potential to shorten the timeframe for an infant to rely on diapers. The subsequent potential for reduced environmental impact further justifies why this integrative review is needed.

Most of the included studies and study participants originate from low and middle-income, non-English-speaking countries. This categorisation does not assume that people practice AITT within these countries primarily because of their income. Caregivers adopt AITT based on cultural traditions and alternative understandings of physiology, such as those informed by Traditional Chinese Medicine (TCM) (Dombroski, 2015) and resource scarcity. The World Bank categorisation based on a country’s Gross Domestic Product (GDP) is utilised in this review as a grouping denominator in line with the World Health Organization (WHO) and The United Nations (UN) nomenclature.

## Methods

### Search Procedure

This integrative review was conducted according to PRISMA guidelines (Page et al., 2021) from May to August 2023. Details of the protocol for this review were registered on PROSPERO ID CRD42023452610. Studies were identified through the following six databases: Scopus, MEDLINE, CINAHL, Web of Science, PsycINFO and Google Scholar. The search terms were assisted-infant-toilet-training OR AITT OR elimination-communication OR ((toilet-train* OR potty-train*) AND (assist* OR infan* OR first-two-years OR birth-to-18-months). A bibliographic search was completed by manually reviewing the reference lists of full-text review studies.

### Selection Criteria

**Fig. 1:**
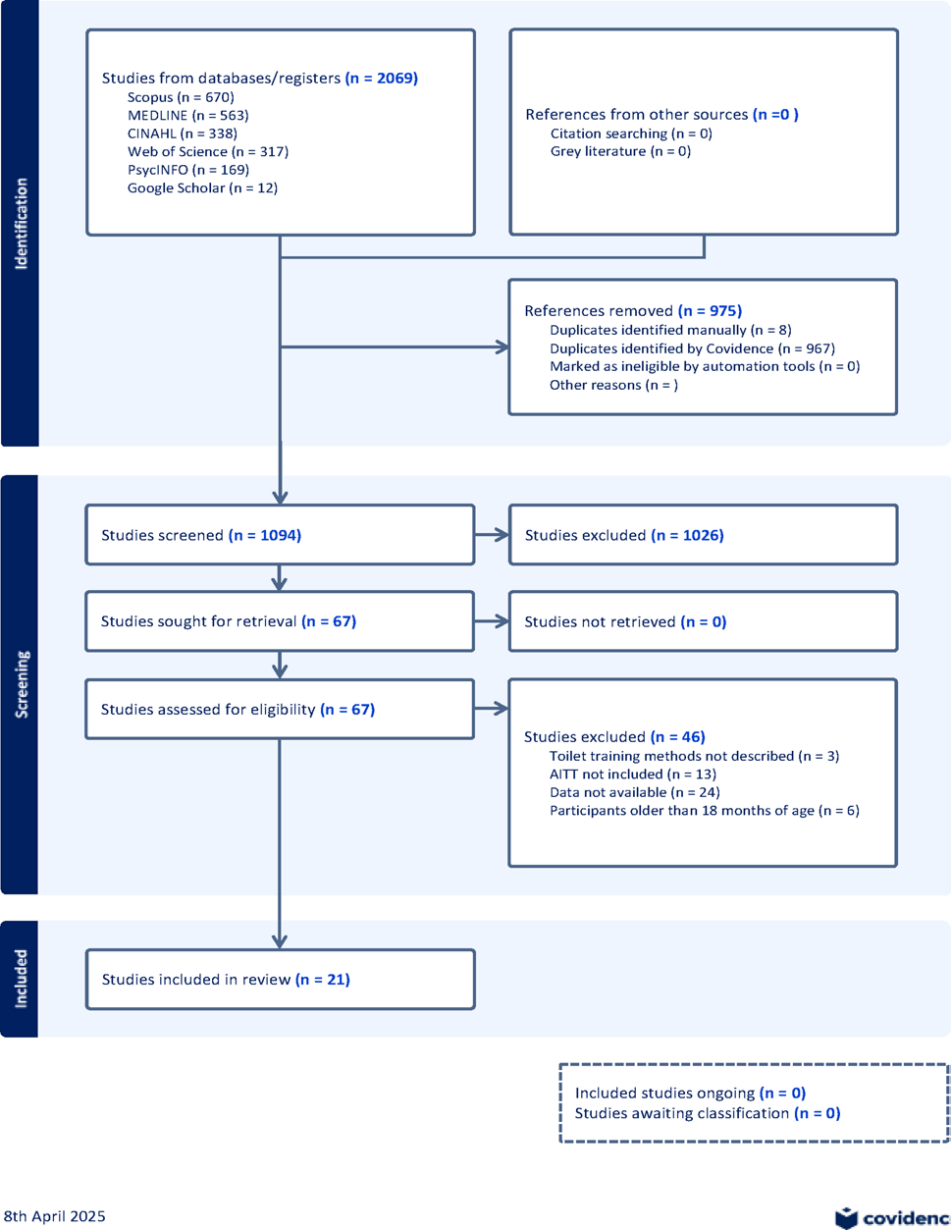
Assisted Infant Toilet Training and Bladder and Bowel Health: A Global Integrative Review

Studies had to meet the following inclusion criteria for the review: (1) to include infants cared for with the AITT or EC method commencing between birth and 18 months of age. (2) Published in a peer-reviewed journal. Studies were excluded from this integrative review for the following reasons: (1) opinion pieces, (2) studies without qualitative or quantitative data collection, (3) studies that did not mention AITT or EC were practised in at least part of the study population. No date range or language restrictions were specified. The search strategy yielded a total of 1094 studies after removing duplicates. The PRISMA flow diagram in Figure 1 presents the number of records considered at each review stage. One author performed the searches, and two others screened the studies independently. Disagreements in selection were resolved by group discussion.

### Methodological Quality Assessment

Methodological Quality Assessment was conducted using two tools: JBI critical appraisal checklists (Moola, 2020) were used to assess the trustworthiness and relevance of all studies included in the review. Additionally, GRADE (Grading of Recommendations, Assessment, Development, and Evaluations) software (Prime, 2024) was used to evaluate the quality and strength of recommendations, providing a framework to assess the certainty of evidence across various outcomes. Six studies that provided comparative data were assessed for risk of bias, inconsistency, indirectness and imprecision, and the study’s overall risk of bias was assessed accordingly.

### Data Extraction and Integration

The outcomes of the included studies were extracted with the assistance of Covidence software (Innovation, 2023) and compiled in a table. A second author audited the extraction table (Table 1). Extracted data included author and year of publication, study design, number and location of participants, AITT commencement and completion age, bladder and/or bowel dysfunction, and qualitative themes. Given the heterogeneity of the studies, meta-analysis was not applicable. The constructed themes were integrated across studies into four thematic headings.

**Table 1:** International studies on AITT

Author/year	Study design	Participants (*N*)	Population (*P*)	Intervention (I)	Comparison (C)	Initiation/completion ages	BBD/other outcomes
Bender et al. (2021)	Longitudinal study	85	Infants in diverse international settings	AITT	N/A	Median initiation 2.5 m; 13% completed by 12 m	constipation 1–4%
Benjasuwantep and Ruangdaraganon (2011)	Longitudinal study	47	Thai infants	AITT	N/A	5/47 by 4 m; 38/47 by 12 m; 22/45 completed by 12 m	no BBD reported
deVries and deVries (1977)	Ethnographic case study	34	East African infants	AITT from 2–3 weeks	N/A	10/16 completed by 4–6 m 30/34 initiated by 2–3w	no BBD reported
Dombroski (2016)	Qualitative research	-	EC practitioners in Australia and NZ	EC	N/A	-	Themes: sustainable consumption, shared knowledge, attachment; no BBD reported
Dombroski (2018)	Qualitative research	N/A	EC practitioners in China, Australia and NZ	EC	N/A	-	Themes: intuition, emotional care, redistribution of labour; no BBD reported
Duong et al. (2010)	Longitudinal study	47	Vietnamese infants	AITT from birth	Swedish cohort TT after 18 m	33/47 initiated from birth	Post-void residual volume minimal by 9 m vs. after 2 years
Duong et al. (2013a, b)	Qualitative research	47	Vietnamese mothers	AITT from birth	N/A	-	Themes: child communication, developmental patience; no BBD reported
Geist and Bammer-Zimmer (2023)	Longitudinal study	674	Infants in Western Europe	EC	Diaper only, no EC	Completed for defecation median age 14–20 m vs. 29 m for diaper only	No increased BBD reported for EC
Jordan et al. (2020)	Pilot study	10	Canadian infants	EC commenced before 1 month	-	-	Reduction in unexplained infant crying; no BBD reported
Largo et al. (1996)	Longitudinal study	629	Swiss infants (2 cohorts)	AITT from 7 m	TT after 18 months	Initiation 7 m; 20% completion 18–24 m vs. 19–21 m initiation; less than 10% complete by 24 m	no BBD reported
Li et al. (2020)	Case-control study	376	Chinese children	AITT	TT after 18 months	-	PNE 34.6% vs. 58.2%
Rugolotto et al. (2008)	Longitudinal study	286	Infants in diverse international settings	AITT	N/A	75% initiated before 6 m; median 17.4 m daytime completion	constipation < 2%; UI 3.8%; haemorrhoids 2.5%
Smeets et al. (1985)	Case report	4	Dutch infants	AITT from 3 months	N/A	Initiation: 3.1–6.6 m; completion: 12 m	no BBD reported
Solarin et al. (2017)	Cross-sectional study	350	Nigerian children	AITT commenced before 12 m	N/A	142/350 initiated before 12 m	no BBD reported
Sun and Rugolotto (2004a, b)	Case report	1	Chinese Italian family	AITT from 1 m	N/A	Initiation 1 m; Completion by 24 m	no BBD reported
Wang et al. (2019)	Cross-sectional study	18,016	Chinese preschoolers	AITT comenced before 3 months	TT after 24 m	-	PNE 3.4% vs. 14.1%
Xing et al. (2020)	Cross-sectional study	10,133	Chinese children	AITT start time 6–11 months	TT after 18 m	-	OAB 8.5% vs. 13.3%
Xu et al. (2021)	Cross-sectional study	10,166	Chinese children	AITT start time 0–12 months	TT after 18 m	-	BBD 1.4% vs. 15.7%
Yang et al. (2011)	Cross-sectional study	235	Taiwanese children	AITT	TT after 18 m	66/235 before 18 m; 90.2% within 6 months for urine	No increase BBD
Yang Jing et al. (2020)	Cross-sectional study	2297	Chinese children	AITT start time before 6 months	TT after 24 m	-	DUI 5.9% vs. 13.0%
Yu et al. (2022)	Cross-sectional study	11,090	Chinese preschool-aged children	AITT start time before 12 months	TT after 18 m	-	DDD 2.4% vs. 9.4%

This table summarises study design, participant characteristics, initiation and completion ages, and reported outcomes

### Results of Quality Appraisal

JBI checklists were utilised to appraise all studies included in this review (see Appendix 1). Two reviewers concluded that all included studies were of adequate methodological quality. The seven cross-sectional studies were rigorously designed and reported, confounding factors were identified in six of the seven studies, and the statistical analysis reflected careful analysis with these in mind. None of the six longitudinal cohort studies (Bender et al., 2021; Benjasuwantep & Ruangdaraganon, 2011; Duong et al., 2010; Geist & Bammer-Zimmer, 2023; Largo, 1996); Rugolotto et al., 2008) utilised an unexposed group based on the same population to compare with the exposed group. This consideration decreases the quality and validity of the study findings. The three qualitative studies (Dombroski, 2016, 2018; T. H. Duong et al., 2013a, b) satisfied all JBI quality criteria for a qualitative study. Their contribution provides valuable insights into the motivation and personal experience of people practising this method. These insights provided reasoning as to why AITT may be valuable beyond what is quantifiable. The one case-control study (Li et al., 2020) was high quality and satisfied all JBI appraisal markers. The case studies and pilot study (deVries & deVries, 1977; Jordan et al., 2020; Smeets et al., 1985; Sun & Rugolotto, 2004a, b) were adequately designed and contribute to the body of knowledge on AITT and can help direct future research in this area.

Five cross-sectional and one case-control study from China, investigating various bladder and bowel dysfunctions were subject to GRADE certainty assessment and quality appraisal. Only these six studies were included, as their methodology and data collection were sufficient for GRADE analysis. All six were deemed at serious risk of bias due to their non-randomised designs, which inherently limit control over unmeasured confounding factors. While the studies were well-designed, adjusted for multiple confounders, and statistically sound, they could not be rated as having a low risk of bias. Consequently, the certainty of evidence was rated as very low to moderate; three studies were upgraded due to large effect sizes and precise estimates.

## Results

Twenty-one studies were included in the final analysis. Table 1 presents the characteristics of the included studies. Six studies with a comparison group were entered into the GRADE evidence profile (see Table 2).

**Table 2:** GRADE evidence profile for AITT

Certainty assessment							No of patients		Effect		Certainty	Importance
№ of studies	Study design	Risk of bias	Inconsistency	Indirectness	Imprecision	Other considerations	Assisted Infant Toilet Training (AITT)	other methods	Relative(95% CI)	Absolute(95% CI)		
Xing et al. 2020- Overactive bladder: AITT start time 6-11 months compared to no AITT (assessed with: self-administered questionnaire)												
1	Non-randomised studies	Serious^a^	Not serious	Not serious	Not serious	None	260/3057 (8.5%)	60/452 (13.3%)	**OR 0.606**(0.446 to 0.824)	**48 fewer per 1,000**(from 69 fewer to 21 fewer)	Low	IMPORTANT
Xu et al. 2021- Bladder and bowel dysfunction: AITT start time 0- 12 months compared to no AITT (assessed with: self-administered anonymous questionnaire)												
1	Non-randomised studies	Serious^a^	Not serious	Not serious	Not serious	None	66/4870 (1.4%)	101/643 (15.7%)	**OR 0.071**(0.050 to 0.102)	**144 fewer per 1,000**(from 148 fewer to 138 fewer)	Moderate b	IMPORTANT
Yang et al.^ 2020^- Daytime urinary incontinence: AITT start time before 6 months compared to after 24 months (assessed with: questionnaires for parents)												
1	Non-randomised studies	Serious^a^	Not serious	Not serious	Serious	None	28/471 (5.9%)	30/231 (13.0%)	**OR 0.560**(0.321 to 0.979)	**53 fewer per 1,000**(from 84 fewer to 2 fewer)	Very low	IMPORTANT
Yu et al. 2022- Disposable diaper dependence: AITT start time before 12 months compared to no AITT (assessed with: anonymous questionnaire)												
1	Non-randomised studies	Serious^a^	Not serious	Not serious	Not serious	None	177/7471 (2.4%)	116/1234 (9.4%)	**OR 0.234**(0.184 to 0.298)	**70 fewer per 1,000**(from 75 fewer to 64 fewer)	Moderate c	IMPORTANT
Wang et al. 2019- Primary nocturnal enuresis: AITT start time before 3 months compared to after 24 months (assessed with: anonymous self-administered questionnaires)												
1	Non-randomised studies	Serious^a^	Not serious	Not serious	Not serious	None	129/3756 (3.4%)	311/2203 (14.1%)	**OR 0.293**(0.234 to 0.368)	**95 fewer per 1,000**(from 104 fewer to 84 fewer)	Moderate d	IMPORTANT
Li et al. 2020– Primary nocturnal enuresis: AITT/EC vs. no AITT/EC (assessed with: parent questionnaires)												
1	Non-randomised studies	Serious ^a^	Not serious	Not serious	Not serious	None	157/454 (34.6%)	219/376 (58.2%)	**OR 1.08 (1.04 to 1.12)**	**17 fewer per 1,000 (from 8 fewer to 26 fewer)**	Low	IMPORTANT

This table presents the certainty of evidence and effect estimates for AITT on bladder and bowel health outcomes compared to no AITT, based on non-randomised studies

Outcomes rated in bold under the “Importance” column indicate outcomes judged to be important for decision-making according to the GRADE approach

*CI* confidence interva, *OR* odds ratio

Explanations

^a^Serious risk of bias due to self-report and observational design

^b^ Upgraded due to very large effect size despite risk of bias (Xu et al.)

^c^ Upgraded due to large and precise effect size (Yu et al.)

^d^ Upgraded due to large and precise effect size (Wang et al.)

### Study Designs

Seven studies were cross-sectional studies (Solarin et al., 2017; Wang et al., 2019; Xing et al., 2020; Xu et al., 2021; Yang, 2020; Yang et al., 2011; Yu et al., 2022), six were longitudinal cohort studies (Bender et al., 2021; Benjasuwantep & Ruangdaraganon, 2011; Duong et al., 2010; Geist & Bammer-Zimmer, 2023; Largo, 1996); Rugolotto et al., 2008), four were case reports or pilot studies (deVries & deVries, 1977; Jordan et al., 2020; Smeets et al., 1985; M. I. N. Sun & Rugolotto, 2004a, b) three were qualitative studies (Dombroski, 2016, 2018; T. H. Duong et al., 2013a, b), and a single case-control study (Li et al., 2020).

### Participants

The twenty-one studies included a total of 54 527 participants. The number of participants ranged from 1 (Sun & Rugolotto, 2004a, b) to 18 016 (Wang et al., 2019).

### Themes

Four themes were synthesised across the twenty-one included studies.

### Commencement and Completion Age

Parents and caregivers in all studies initiated AITT between birth and 18 months before the infant showed signs of readiness for an independence-focused toilet training method. In Duong’s qualitative study (T. H. Duong et al., 2013a, b) from Vietnam, most mothers with newborns had commenced AITT. “*I often help my child to pee at certain times by using a whistling sound*,* after feeding and when he wakes up”* (Duong et al., 2013a, b, p. 810). The AITT completion age range in this review was reported as being between four to six months (deVries & deVries, 1977) to thirty months (Yang et al., 2011). Most infants in Duong’s study were having very few accidents by eighteen months of age and becoming independent with the toileting process; one mother states, “*Before peeing*,* he takes off his pants and sits on the potty. If I am here to help him*,* he lets me do that; if not*,* he manages by himself*” (T. H. Duong et al., 2013a, b, p. 812). Toilet training was regarded as complete despite the infant needing caregiver assistance with some aspects of the toileting process (T. H. Duong et al., 2013a, b). The definition of completion age varied widely across the studies. Daytime dryness, night-time dryness, and bladder and/or bowel control were some or all components of this definition.

### Bowel and or Bladder Dysfunction

The GRADE evidence profile (Prime, 2024) displays bladder and bowel dysfunction outcomes (See Table 2). In six studies from China, AITT showed a potential protective effect on various bladder or bowel dysfunctions. Xing’s team (Xing et al., 2020) showed a reduction in Overactive Bladder (OAB) rates in children when AITT was practised, mainly before 12 months of age. OAB is defined as urgency and increased daytime frequency with or without urinary incontinence that is not due to pathological or neurological factors (Xing et al., 2020). Xing’s study showed a protective effect when AITT was commenced before 12 months of age, with OAB rates at 8.5%, compared to 13.3% when toilet training commenced after 18 months (Xing et al., 2020). This is 48 fewer diagnoses of OAB per 1000 children.

Bladder and Bowel Dysfunction (BBD), which describes the co-existence of voiding dysfunction symptoms and functional constipation and/or faecal incontinence, was less common in children who commenced AITT before twelve months of age compared to those who started toilet training from twenty-four months of age (*P* < 0.001) (Xu et al., 2021). BBD incidence for those who commenced AITT before 12 months of age was 1.4% and increased to 15.7% when AITT was delayed until after 25 months (Xu et al., 2021).

Daytime urinary incontinence (DUI) also saw a reduction in incidence in Yang’s trial (Yang, 2020). The International Paediatric Urinary Continence Association defines DUI as the involuntary leakage of urine through the urethra during the day, a common symptom during the development of urinary control in young children (Yang, 2020). Kindergarten children who commenced AITT before 6 months of age had a DUI rate of 5.9%; when toilet training was commenced after 18 months, the incidence increased to 13% (Yang, 2020). This translates to 53 fewer per 1000 kindergarten children experiencing DUI when AITT commences before 6 months.

The number of children with Disposable Diaper Dependence (DDD) was reduced with the presence of AITT (Yu et al., 2022). Disposable Diaper Dependence is defined as disposable diaper use since birth and heavy dependence on DDs after the age of 2 years; crying, resisting and refusing to cooperate with parental instructions; an inability to control urination and defecation when a DD is not applied; and a restored calm state after DD application (Yu et al., 2022). In Yu’s study (2022), AITT practice before 12 months of age appeared to be a protective factor against DDD, showing a 7% reduction in the incidence or 70 fewer diagnoses per 1000 children compared to infants who commenced toilet training after 18 months of age (Yu et al., 2022).

Primary nocturnal enuresis (PNE) is diagnosed when children over 5 years of age have at least one enuresis episode per month, lasting at least 3 months (Wang et al., 2019). Li et al. (Li et al., 2020) and Wang’s trial (Wang et al., 2019) both showed a decrease in the incidence of PNE in children when AITT was practised. In Wang’s study, infants who commenced AITT before 3 months of age had an incidence of PNE of 3.4%, when toilet training was initiated after 24 months of age, the rate increased significantly to 14.1% (Wang et al., 2019). This is 95 fewer diagnoses of PNE per 1000 infants. In the case-control study from Li et al., children whose caregivers implemented AITT had a lower risk of PNE with 17 fewer cases per 1000 children (Li et al., 2020) compared to those who started toilet training after 18 months.

Three studies that reported on rates of constipation while practising AITT found occurrence rates were low and not more significant than the average occurrence rate in infants, which varies widely between 1 and 25.8% (Bender et al., 2021; Rugolotto et al., 2008; Yang et al., 2011). A longitudinal study by Duong et al. (Duong et al., 2010) suggests that AITT can help develop an infant’s bladder capacity and promote complete emptying. In this study, infants cared for with AITT methods showed a minimal post-void residual volume by nine months compared to a Swedish population who achieved this after 2 years of age (Duong et al., 2013a, b). Residual amounts of urine in the bladder can be a risk factor for Urinary Tract Infections (UTI) (Duong et al., 2010).

### Signals and Timing

The method in which AITT is practised has been reported in thirteen studies. Eleven of these studies mention that responding to an infant’s communication signals for elimination is a key component of the AITT method. Between 15.2% and 90% of infants rely on signals to convey their elimination needs. Dombroski’s research, using qualitative, ethnographic data from an Australian and New Zealand online parenting forum, highlights that communication and healthy attachment are two of the goals of AITT (Dombroski, 2016). Dombroski theorises that AITT helps develop connection and intuition regarding reading an infant’s needs (Dombroski, 2018). Interpreting and responding to an infant’s verbal and non-verbal signals and following natural timing and intuition can all be components of AITT practice. Jordan and colleagues (Jordan, 2014) theorise that what we diagnose as infant colic, which is defined as unexplained infant crying, is the infant communicating an unmet need for elimination support. Jordan’s pilot study (Jordan et al., 2020) provides provisional support for the theory that AITT practice reduces rates of unexplained infant crying.

### Diapers

The use of diapers was mentioned in fourteen of the twenty-one studies in this review. The Vietnamese studies noted that diapers were not used frequently during the day and sometimes at night (T. H. Duong et al. 2010, 2013a, b). The Nigerian study showed a preference for disposable diapers compared to cloth, 90% compared to 8% respectively (Solarin et al., 2017). Geist’s study from Austria showed that the more frequent diaper-free times infants had during the day, the earlier they were toilet trained and the more regular their defecation pattern was (Geist & Bammer-Zimmer, 2023). Furthermore, using cloth compared to disposable diapers significantly lowered the toilet training age in this study (Geist & Bammer-Zimmer, 2023). Both cloth and disposable diapers are utilised while AITT is practised. AITT can reduce the timeframe and number of diapers an infant utilises. The use of both reusable and disposable diapers has a significant impact on our environment (Holdway, 2023). The average disposable diaper results in a global warming impact of approximately 550 kg of carbon dioxide over the two-and-a-half years an infant is typically wearing diapers (Holdway, 2023).

## Discussion

The primary outcome of this integrative review is that multiple observational studies show the potential protective effect of AITT on various bladder and bowel dysfunctions. However, these results are based on non-randomised studies and are at *serious risk of bias* as there is no control group. As outlined in the GRADE Summary of Findings (Table 2), the certainty of evidence ranged from low to moderate due to the observational nature of the included studies and the associated risk of bias. However, several studies demonstrated large and precise effect sizes, warranting an upgrade in confidence. Future prospective research in high-income countries with large sample sizes and randomised groups is warranted to test this method’s efficacy and provide experiential data. A randomised, two-armed intervention study comparing AITT with conventional toilet training is underway in Sweden (Nilsson et al., 2022). Studying a culturally embedded and socially facilitated practice such as AITT within a randomised controlled trial presents inherent difficulties. It is not easy to separate the physical practice from the culture, society and environment that gave rise to it. It could be questioned if the RCT, despite being the gold standard of evidence, is the most appropriate method to study AITT. Despite the inherent risk of confounding and selection bias, large, well-designed observational studies may be a sound study design to examine AITT practice and its outcomes.

This integrative review highlights that omitting AITT from prominent toilet training guidelines in high-income countries is unjustified. The evidence of an absence of increased bladder and bowel dysfunction compared to toilet training after 18 months of age supports the safety of this method for infants. Demonstrated safety combined with the potential to reduce their ecological footprint, parents and caregivers should be informed about all toilet training options to choose a method that suits their lifestyle and values.

An infant’s elimination signals are not well recognised in many cultures where AITT is uncommon but can play a key role in the AITT method. These signals can be subtle or conspicuous; responding to them promptly and appropriately is a key component of infant well-being. As AITT often relies on developing an infant’s verbal and non-verbal communication signals, the potential for this method to enhance caregiver-infant interaction and reduce rates of *infant colic* is another avenue for future research.

## Limitations

The literature search was limited to published journal articles, and a meta-analysis was not undertaken. No language restrictions were used in the initial search; however, non-English studies may have been missed as only English keywords were used. Most AITT studies originate from low—and middle-income, non-English-speaking countries. Furthermore, the literature available was limited to retrospective observational studies, and no completed randomised trials were identified. Future studies from high-income countries will help further assess this method’s generalisability across cultural contexts.

## Conclusions

AITT is a toilet training method that does not rely on the infant’s developmental readiness. As such, it cannot be directly compared with child-orientated methods. This review identifies multiple potential benefits of AITT for infants, including reduced risk of bladder and bowel dysfunction and environmental impact. However, this evidence for these health benefits is at *serious risk of bias*. Importantly, no comparative studies in this review demonstrate that AITT is associated with more risk than toilet training after 18 months. Omitting AITT from nationally recognised toilet training guidelines in high-income countries appears unjustified. The initiation and completion age when using this method is often 18–24 months before independence-focused toilet training commences; there is a potential reduction in the number of diapers utilised over the toilet training period. The potential for AITT to enable a decreased ecological footprint should be investigated further.

## Appendix

**Table:**

JBI critical appraisal checklist for qualitative research	Dombroski (2016)	Dombroski (2018)	Duong et al. (2013a, b)
Is there congruity between the stated philosophical perspective and the research methodology?	Y	Y	Y
Is there congruity between the research methodology and the research question or objectives?	Y	Y	Y
Is there congruity between the research methodology and the methods used to collect data?	Y	Y	Y
Is there congruity between the research methodology and the interpretation of results?	Y	Y	Y
Is there a statement locating the researcher culturally or theoretically?	Y	Y	Y
Is the influence of the researcher on the research, and vice- versa, addressed?	Y	Y	Y
Are participants, and their voices, adequately represented?	Y	Y	Y
Is the research ethical according to current criteria or, for recent studies, and is there evidence of ethical approval by an appropriate body?	Y	Y	Y
Do the conclusions drawn in the research report flow from the analysis, or interpretation, of the data?	Y	Y	Y

**Table:**

JBI critical appraisal checklist for cohort studies	Bender et al. (2021)	Benjasuwantep and Ruangdaraganon (2011)	Duong et al. (2010)	Geist and Bammer-Zimmer (2023)	Largo et al. (1996)	Rugolotto et al. (2008)	Jordan et al. (2020)
Were the two groups similar and recruited from the same population?	N/A	N/A	N/A	N/A	Y	N/A	N/A
Were the exposures measured similarly to assign people to both exposed and unexposed groups?	N/A	N/A	N/A	N/A	Y	N/A	N/A
Was the exposure measured in a valid and reliable way?	Y	Y	Y	Y	Y	Y	Y
Were confounding factors identified?	Y	Y	Y	N	Y	N	Y
Were strategies to deal with confounding factors stated?	N	N	Y	N	N	N/A	Y
Were the groups/participants free of the outcome at the start of the study (or at the moment of exposure)?	Y	Y	Y	Y	Y	Y	Y
Were the outcomes measured in a valid and reliable way?	Y	Y	Y	Y	Y	Y	Y
Was the follow up time reported and sufficient to be long enough for outcomes to occur?	Y	Y	Y	Y	Y	Y	Y
Was follow up complete, and if not, were the reasons to loss to follow up described and explored?	Y	Y	Y	N/A	N	Y	Y
Were strategies to address incomplete follow up utilized?	Y	U	Y	N/A	N/A	Y	Y
Was appropriate statistical analysis used?	Y	Y	Y	Y	Y	Y	Y

**Table:**

JBI critical appraisal checklist for cross sectional studies	Solarin et al. (2017)	Wang et al. (2019)	Xing et al. (2020)	Xu et al. (2021)	Yang et al. (2011)	Yang et al. (2020)	Yu et al. (2022)
Were the criteria for inclusion in the sample clearly defined?	Y	Y	Y	Y	Y	Y	Y
Were the study subjects and the setting described in detail?	Y	Y	Y	Y	Y	Y	Y
Was the exposure measured in a valid and reliable way?	Y	Y	Y	Y	Y	Y	Y
Were objective, standard criteria used for measurement of the condition?	Y	Y	Y	Y	Y	Y	Y
Were confounding factors identified?	Y	Y	Y	Y	N	Y	Y
Were strategies to deal with confounding factors stated?	Y	Y	Y	Y	N	Y	Y
Were the outcomes measured in a valid and reliable way?	Y	Y	Y	Y	Y	Y	Y
Was appropriate statistical analysis used?	Y	Y	Y	Y	Y	Y	Y

**Table:**

JBI critical appraisal checklist for case reports	de Vries & de Vries (1977)	Sun and Rugolotto (2004a, b)	Smeets et al. (1985)
Were patient’s demographic characteristics clearly described?	Y	Y	Y
Was the patient’s history clearly described and presented as a timeline?	N/A	Y	Y
Was the current clinical condition of the patient on presentation clearly described?	Y	Y	Y
Were diagnostic tests or assessment methods and the results clearly described?	N/A	Y	Y
Was the intervention(s) or treatment procedure(s) clearly described?	Y	Y	Y
Was the post-intervention clinical condition clearly described?	Y	Y	Y
Were adverse events (harms) or unanticipated events identified and described?	Y	Y	N
Does the case report provide takeaway lessons?	Y	Y	Y

**Table:**

JBI critical appraisal checklist for case control studies	Li et al. (2020)
Were the groups comparable other than the presence of disease in cases or the absence of disease in controls?	Y
Were cases and controls matched appropriately?	Y
Were the same criteria used for identification of cases and controls?	Y
Was exposure measured in a standard, valid and reliable way?	Y
Was exposure measured in the same way for cases and controls?	Y
Were confounding factors identified?	Y
Were strategies to deal with confounding factors stated?	Y
Were outcomes assessed in a standard, valid and reliable way for cases and controls?	Y
Was the exposure period of interest long enough to be meaningful?	Y
Was appropriate statistical analysis used?	Y

Key

*Y* Yes

* N* No

* U*Unclear

*N/A* Not applicable

## References

[CR1] Accardo, P. (2006). Whos training whom? *The Journal of Pediatrics*, *149*(2), 151–152. 10.1016/j.jpeds.2006.04.026 16887422 10.1016/j.jpeds.2006.04.026

[CR2] Bakker, E., & Wyndaele, J. J. (2000). Changes in the toilet training of children during the last 60 years: The cause of an increase in lower urinary tract dysfunction? *BJU International,**86*(3), 248–252. 10.1046/j.1464-410x.2000.00737.x 10930924 10.1046/j.1464-410x.2000.00737.x

[CR3] Bender, J. M., Lee, Y., Ryoo, J. H., Boucke, L., Sun, M., Ball, T. S., Rugolotto, S., & She, R. C. (2021). A longitudinal study of assisted infant toilet training during the first year of life. *Journal of Developmental & Behavioral Pediatrics*, *42*(8), 648–655. 10.1097/dbp.0000000000000936 34618722 10.1097/DBP.0000000000000936

[CR4] Benjasuwantep, B., & Ruangdaraganon, N. (2011). Infant toilet training in Thailand: Starting and completion age and factors determining them. *Journal of the Medical Association of Thailand = Chotmaihet Thangphaet,**94*, 1441–1446. 22295729

[CR5] Brazelton, T. B. (1962). A child-orientated approach to toilet training. *Pediatrics*, *29*(1), 121–128. 10.1542/peds.29.1.121 13872676

[CR6] de Carvalho Mrad, F. C., da Silva, M. E., Moreira Lima, E., Bessa, A. L., de Bessa Junior, J., Netto, J. M. B., & de Almeida Vasconcelos, M. M. (2021). Toilet training methods in children with normal neuropsychomotor development: A systematic review. *Journal of Pediatric Urology*, *17*(5), 635–643. 10.1016/j.jpurol.2021.05.010 34090792 10.1016/j.jpurol.2021.05.010

[CR7] deVries, M. W., & deVries, M. R. (1977). Cultural relativity of toilet training readiness: A perspective from East Africa. *Pediatrics*, *60*(2), 170–177. 10.1542/peds.60.2.170 887331

[CR8] Dombroski, K. (2015). Multiplying possibilities: A postdevelopment approach to hygiene and sanitation in Northwest China. *Asia Pacific Viewpoint*, *56*(3), 321–334. 10.1111/apv.12078

[CR9] Dombroski, K. (2016). Hybrid activist collectives: Reframing mothers’ environmental and caring labour. *International Journal of Sociology and Social Policy*, *36*, 629–646. 10.1108/IJSSP-12-2015-0150

[CR10] Dombroski, K. (2018). Learning to be affected: Maternal connection, intuition and elimination communication. *Emotion, Space and Society,**26*, 72–79.

[CR11] Duong, T. H., Jansson, U. B., Holmdahl, G., Sillén, U., & Hellstrom, A. L. (2010). Development of bladder control in the first year of life in children who are potty trained early. *Journal of Pediatric Urology*, *6*(5), 501–505. 10.1016/j.jpurol.2009.11.002 19939737 10.1016/j.jpurol.2009.11.002

[CR12] Duong, T. H., Jansson, U. B., & Hellström, A. L. (2013a). Vietnamese mothers’ experiences with potty training procedure for children from birth to 2 years of age. *Journal of Pediatric Urology,**9*(6, Part A), 808–814. 10.1016/j.jpurol.2012.10.023 23182948 10.1016/j.jpurol.2012.10.023

[CR13] Duong, T., Jansson, U. B., Holmdahl, G., Sillén, U., & Hellström, A. L. (2013b). Urinary bladder control during the first 3 years of life in healthy children in Vietnam - A comparison study with Swedish children. *Journal of Pediatric Urology*. 10.1016/j.jpurol.2013.04.022 23759503 10.1016/j.jpurol.2013.04.022

[CR14] Geist, B. K., & Bammer-Zimmer, R. (2023). Effects of early toilet training and elimination communication with respect to diaper types. *Clinical Pediatrics,* , Article 99228221145268. 10.1177/00099228221145268 10.1177/00099228221145268 36852780

[CR15] Holdway, R. D., Mark (2023). Life cycle Assessment (LCA) of disposable and reusable nappies in the UK. (EV0493). F. A. R. A. Defra - Department for Environment.

[CR16] Horn, I. B., Brenner, R., Rao, M., & Cheng, T. L. (2006). Beliefs about the appropriate age for initiating toilet training: Are there racial and socioeconomic differences? *The Journal of Pediatrics,**149*(2), 165–168. 10.1016/j.jpeds.2006.03.004 16887427 10.1016/j.jpeds.2006.03.004

[CR17] Innovation, V. H. (2023). Covidence Systematic Review Software.

[CR18] Jordan, G. J. (2014). Elimination communication as colic therapy. *Medical Hypotheses*, *83*(3), 282–285. 24962210 10.1016/j.mehy.2014.05.018

[CR19] Jordan, G. J., Arbeau, K., McFarland, D., Ireland, K., & Richardson, A. (2020). Elimination communication contributes to a reduction in unexplained infant crying. *Medical Hypotheses*, *142*, 109811. 10.1016/j.mehy.2020.109811 32422498 10.1016/j.mehy.2020.109811

[CR20] Kiddoo, D. A. (2012). Toilet training children: When to start and how to train. *Canadian Medical Association Journal,**184*(5), 511–512. 10.1503/cmaj.110830 21825046 10.1503/cmaj.110830 PMC3307553

[CR21] Largo, R. M., von Siebenthal, L., & Wolfensberger, K., U (1996). Does a profound change in toilet-training affect development of bowel and bladder control? *Developmental Medicine and Child Neurology*, *38*, 1106–1116. 8973296 10.1111/j.1469-8749.1996.tb15074.x

[CR22] Li, X., Wen, J. G., Shen, T., Yang, X. Q., Peng, S. X., Wang, X. Z., Xie, H., Wu, X. D., & Du, Y. K. (2020). Disposable diaper overuse is associated with primary enuresis in children. *Scientific Reports*, *10*(1), 14407. 10.1038/s41598-020-70195-8 32873840 10.1038/s41598-020-70195-8 PMC7462848

[CR23] Moola, S. M., Tufanaru, Z., Aromataris, C., Sears, E., Sfetcu, K., Currie, R., Qureshi, M., Mattis, R., Lisy, P., & Mu, K., P-F (2020). In E. Aromataris (Ed.), JBI manual for evidence synthesis. M. Z. https://synthesismanual.jbi.global JBI.

[CR24] Network, R. C. (2023). 03/05/2023). Toilet training: a practical guide. Retrieved March 3, 2020 from https://raisingchildren.net.au/preschoolers/health-daily-care/toileting/toilet-training-guide

[CR25] Nilsson, T., Leijon, A., Sillén, U., Hellström, A. L., & Skogman, B. H. (2022). Bowel and bladder function in infant toilet training (BABITT) - Protocol for a randomized, two-armed intervention study. *BMC Pediatrics,**22*(1), 294. 10.1186/s12887-022-03355-6 35590259 10.1186/s12887-022-03355-6 PMC9118841

[CR26] Page, M. J., McKenzie, J. E., Bossuyt, P. M., Boutron, I., Hoffmann, T. C., Mulrow, C. D., Shamseer, L., Tetzlaff, J. M., Akl, E. A., Brennan, S. E., Chou, R., Glanville, J., Grimshaw, J. M., Hróbjartsson, A., Lalu, M. M., Li, T., Loder, E. W., Mayo-Wilson, E., McDonald, S., & Moher, D. (2021). The PRISMA 2020 statement: an updated guideline for reporting systematic reviews. *BMJ*. 10.1136/bmj.n71 33782057 10.1136/bmj.n71 PMC8005924

[CR27] Pediatrics, A. A. (2022). o. Toilet training. *Pediatric Patient Education*. 10.1542/peo_document105

[CR28] Prime, M. U. E. (2024). GRADEpro GDT. In: gradepro.org.

[CR29] Rugolotto, S., Sun, M., Boucke, L., Calò, D. G., & Tatò, L. (2008). Toilet training started during the first year of life: A report on elimination signals, stool toileting refusal and completion age. *Minerva Pediatrica*, *60*(1), 27–35. 18277362

[CR30] Smeets, P. M., Lancioni, G. E., Ball, T. S., & Oltva, D. S. (1985). Shaping self-initiated toileting in infants. *Journal of Applied Behavior Analysis*, *18*(4), 303–308. 10.1901/jaba.1985.18-303 4086412 10.1901/jaba.1985.18-303 PMC1308025

[CR31] Solarin, A. U., Olutekunbi, O. A., Madise-Wobo, A. D., & Senbanjo, I. (2017). Toilet training practices in Nigerian children. *South African Journal of Child Health,**11*, 122–128.

[CR32] Sun, M., & Rugolotto, S. (2004a). Assisted infant toilet training in a western family setting. *Journal of Developmental and Behavioral Pediatrics,**25*(2), 99–101. 10.1097/00004703-200404000-00004 15083131 10.1097/00004703-200404000-00004

[CR33] Sun, M. I. N., & Rugolotto, S. (2004b). Assisted infant toilet training in a western family setting. *Journal of Developmental & Behavioral Pediatrics*. 10.1097/00004703-200404000-00004 10.1097/00004703-200404000-00004 15083131

[CR34] Wang, X. Z., Wen, Y. B., Shang, X. P., Wang, Y. H., Li, Y. W., Li, T. F., Li, S. L., Yang, J., Liu, Y. J., Lou, X. P., Zhou, W., Li, X., Zhang, J. J., Song, C. P., Jorgensen, C. S., Rittig, S., Bauer, S., Mosiello, G., Wang, Q. W., & Wen, J. G. (2019). The influence of delay elimination communication on the prevalence of primary nocturnal enuresis-a survey from Mainland China. *Neurourology and Urodynamics*, *38*(5), 1423–1429. 10.1002/nau.24002 30998267 10.1002/nau.24002

[CR35] Whittemore, R., & Knafl, K. (2005). The integrative review: Updated methodology. *Journal of Advanced Nursing*, *52*(5), 546–553. 10.1111/j.1365-2648.2005.03621.x 16268861 10.1111/j.1365-2648.2005.03621.x

[CR36] Wu, H. Y. (2010). Achieving urinary continence in children. *Nature Reviews Urology*, *7*(7), 371–377. 10.1038/nrurol.2010.78 20531385 10.1038/nrurol.2010.78

[CR37] Xing, D., Wang, Y. H., Wen, Y. B., Li, Q., Feng, J. J., Wu, J. W., Jia, Z. M., Yang, J., Sihoe, J. D., Song, C. P., Hu, H. J., Franco, I., & Wen, J. G. (2020). Prevalence and risk factors of overactive bladder in Chinese children: A population-based study. *Neurourology and Urodynamics,**39*(2), 688–694. 10.1002/nau.24251 31804751 10.1002/nau.24251

[CR38] Xu, P. C., Wang, Y. H., Meng, Q. J., Wen, Y. B., Yang, J., Wang, X. Z., Chen, Y., He, Y. L., Wang, Q. W., Wang, Y., Cui, L. G., Sihoe, J. D., Franco, I., Lang, J. H., & Wen, J. G. (2021). Delayed elimination communication on the prevalence of children’s bladder and bowel dysfunction. *Scientific Reports,**11*(1), Article 12366. 10.1038/s41598-021-91704-3 34117301 10.1038/s41598-021-91704-3 PMC8196082

[CR39] Yang, J. X., Wen, D., Wang, J., Li, Y., Wang, Y., Wang, Q., Hu, Y., Liang, H., Song, Y., Zhao, C., & Xiao-ping, L. B. (2020). The effects of disposable diaper and elimination communication on daytime urination incontinence in children aged 2 to 3 years. *Chinese Journal of Nursing Education,**12*, 1119–1123. 10.3761/j.issn.1672-9234.2020.12.013

[CR40] Yang, S., Zhao, L. L., & Chang (2011). Early initiation of toilet training for urine was associated with early urinary continence and does not appear to be associated with bladder dysfunction. *Neurourology and Urodynamics*, *30*, 1253–1257. 10.1002/nau.20982 21394761 10.1002/nau.20982

[CR41] Yu, J. T., Mao, Q. F., Ji, F. P., Zhao, Y., Hu, H. J., Zhang, Y. P., Yang, J., Wang, Q. W., Lu, W., & Wen, J. G. (2022). Delayed elimination communication is a crucial factor in disposable diaper dependence in Chinese preschool-aged children. *Frontiers in Pediatrics*, *10*, 1053118. 10.3389/fped.2022.1053118 36699294 10.3389/fped.2022.1053118 PMC9869372

